# Dental Legislation

**Published:** 1899-09

**Authors:** Frederick A. Stevenson

**Affiliations:** Montreal, Canada


### DENTAL LEGISLATION.[1]

BY FREDERICK A. STEVENSON, D.M.D., MONTREAL, CANADA.

In discussing the subject of dental legislation, I have thought it best to speak of that with which I am most familiar,—namely, the dental law and its enforcement in the Province of Quebec, Canada.

There are no doubt many who do not see any necessity for interfering with the liberty of the individual to the extent of insisting upon certain requirements being fulfilled before allowing him to practise dentistry. They claim that legislation has only prevented progress and not aided it, and point to those places where dental legislation has been in force the longest as examples of their contention. It will be found, I believe, upon investigation, that when dental law exists together with a low standard of skill, the fault is due to the lack of enforcement of the existing law. Take the Province of Quebec for example, there we have had a stringent dental law since 1869, and we have not many dentists (about two hundred, I think, nearly all of whom are trying to make a living in the city of Montreal). The standard of dental skill in the Province is probably lower on the average than on the rest of this continent. The backward state of dentistry there is due to the timidity or, which is the same thing, the conservative methods of the leading men in the profession. They were reluctant to establish a college of dentistry for fear that there would be so many dentists turned out in the course of a few years that no one would be able to make a living. About five or six years ago, when there was an agitation among some of the younger men in favor of organizing a dental college, one of these worthies came back from a commencement at the dental college in Toronto, where he had been to make a speech, and brought a tale of woe to the effect that there were so many dentists in Toronto that fees had gone to pieces. Men were filling teeth with gold for one dollar, and for amalgam fillings they received only fifty cents. The dentists there were glad to combine a little life insurance business with the practice of dentistry, in order to eke out a precarious existence. There are prob-

---

[1] A paper read before the Alumni of the Harvard Dental School, on "Alumni Day," June 26, 1899.

ably men in Boston to-day who are receiving fees on about the same scale as those above mentioned. I know that there are in Montreal, and they have been there ever since I have known anything about dentistry in the city. All this, if rightly judged, is not an argument against raising the standard of dental education, but one strongly in its favor. Will a man who has passed a stiff entrance examination and spent three or four years in acquiring the knowledge and skill requisite for the practice of modern dentistry be content with day laborers' wages? I think not.

Another reason brought forward by these very conservative gentlemen, more commendable, perhaps, as it showed the modesty of their estimation of their own acquirements: They claimed that there were no men to be found who were qualified to give lectures or to demonstrate practice. However, in spite of obstruction, instructors were found who were willing to give their time and work for the good of the cause, and we have now a dental faculty who are doing excellent work and who are not too old or set in their ways to adopt suggestions and ideas simply because they are new. The course is given in French as well as in English. The student of dentistry under the old *régime* was under notarial agreement with his preceptors to serve him for four years. He learned what he could in the work-room, mixing plaster of Paris and polishing rubber plates. It followed, as a consequence, that the examining board were afraid to hold a thorough examination, and every man who had faithfully put in his time obtained his license after passing a very easy examination. Of course, many an ignorant and incompetent person was let loose on the public, and we have had periodical doses of quack advertising by these men, which, of course, degrades us socially in the estimation of our sister profession of medicine and in the eyes of the public. Since the establishment of the dental college there has been a great improvement in the classes of students. This has also been greatly helped by the adoption of a higher standard for the preliminary examination which must be passed before the applicant can register as a dental student.

The reason for the backward state of dentistry in the Province of Quebec is, therefore, to be found in the inadequate provision formerly made by the Dental Association of the Province of Quebec, for the education of students, and also in the timidity of the executive board in enforcing the existing dental law.

The most sensible remedy for the state of things above described, it seems to me, would be to have a dental law for the whole of the

Dominion of Canada, instead of each Province legislating for itself.

As it is at present, no graduate from the Dental College of the Province of Quebec can practise in Ontario, and no graduate from the College of Dental Surgery of Ontario can practise in Quebec.

If in the United States there could be a central executive board or college of dentistry, whose duty it should be to establish the regulations and requirements to be fulfilled by every one entering the dental profession, it would have the effect of raising the standard of average efficiency, and tend to draw the whole profession together.

This national college or board could give a license to practise, but need not necessarily confer a degree or be a teaching body. The various colleges already in existence would not feel, in that case, that their privileges were being encroached upon. This national college or board would be an examining body simply; besides examining candidates, it could issue a list of colleges whose graduates would be entitled to practise without further examination.

The question of a preliminary examination is an important one. I can testify to the great improvement at once noticeable in the men who entered dentistry in the Province of Quebec after the adoption of a high standard of general education.

We have not yet adopted the suggestion already talked of in this country,—namely, that of a mechanical examination as part of the preliminary,—but I think it a very valuable suggestion and one which deserves to be carefully worked out.

What we want in dentistry, as in every profession, are men of brains and common sense, and if dental legislation does not help us to that, then the sooner it is discarded the better.

Where dental legislation is to be of real service to the public and the profession it should provide a law, stating the requirements necessary to be fulfilled by persons before being licensed to practise, and should also contain clauses whereby the executive board should be empowered to suspend or confiscate the license of any dentist in active practice who infringed the by-laws enacted for the good of the public and the profession.

The following is a brief summary of the dental law of the Province of Quebec.

1. All licensed dentists practising in this Province are incorporated under the name of Dental Association of the Province of Quebec.

2. Board of Examiners are charged with managing the business of the Association.

3. Number of the members who form the Board and method of election.

4. Powers of the Board defined. They enact by-laws subject to approval of the Association and appoint the faculty of the dental college.

5. Requirements to be fulfilled before entering the examination for license.

6. Requirements to be fulfilled before commencing to study dentistry.

7. Concerning examinations, examiners (4061), and assessors.

8. Complaints before the Board for breaches of discipline and the law for the guidance of the Board in taking action against the offender.

9. Clause exempting licensed physicians of the Province of Quebec from examination except in the subjects of operative and mechanical dentistry proper.

10. Licensed dentists have the exemptions and privileges of physicians.

BY-LAWS.

The preliminary examination:

### BOARD OF EXAMINERS DENTAL ASSOCIATION OF THE PROVINCE OF QUEBEC.

### Programme of the Preliminary Examination for English-Speaking Candidates.

GROUP A.—CLASSICS.

*Latin.*—Cæsar's Commentaries, Books I., II., III. Virgil's Eneid, Books I., II. Questions on Grammar and Construction.

*English.*—Grammar and Analysis. A critical knowledge of one of Shakespeare's plays. "King Lear" for 1899 and 1900. "Hamlet" for 1901 and 1902.

*French.*—Questions on Grammar and Analysis. Translation into English of Extracts from Fenelon's "Aventures de Télémaque." Translation into French of Extracts from Washington Irving's "Life of Columbus." Translation of short English sentences into French and *vice versa.*

*Literature.*—Principles of the subject, with the History of Greek and Roman literature of the classical age, and of English literature from the beginning of the seventeenth century to the present time.

*History.*—Outlines of Greek and Roman History, with a rather more detailed History of England, France, and Canada.

*Geography* modern, especially of Britain and France, and of their colonies and possessions, especially of Canada.

GROUP B.—SCIENCES.

*Arithmetic.*—To the end of Square Root, and a practical knowledge of the Metrical System.

*Algebra.*—To simultaneous equations of the first degree, inclusive.

*Geometry.*—Euclid, Books I., II., III., and the first twenty propositions of Book VI.; also the measurement of the surfaces and volumes of the regular geometrical figures.

*Botany.*—As in Gray's "How Plants Grow."

*Chemistry.*—As in Remsen's "Elements of Chemistry."

*Philosophy.*—Logic, as in Jevon's Logic. Intellectual and Moral Philosophy, as in Professor Murray's Hand-Books.

*Physics.*—Elementary Statics and Dynamics of Solids and Fluids, with the Chapter on Heat, according to Peck's.

N. B.—Candidates must produce certificates of good moral character.

NOTICE.—Candidates may take one Group at one examination and the other Group at the next subsequent examination. Failure in one subject nullifies the success in the whole Group, but failure in one Group does not nullify success in the other. In order to pass, the candidates must obtain sixty per cent. in Latin, English, French, and Arithmetic, and fifty per cent. in the other subjects.

The examinations are held at Montreal, on the first Wednesday in April and October. Applications to be made in person to the Secretary, accompanied with the receipt of the Treasurer for matriculation fee, at least ten days before the date of examination. Fee, $20. Should the candidate be unsuccessful, one-half of the fee will be returned.

DR. E. B. IBBOTSON, *President.*
DR. EUDORE DUBEAU, *Secretary.*
DR. F. A. STEVENSON, *Treasurer.*
DR. W. J. KERR, *Registrar.*

This, as you will see, insures a good general education to begin with, if the candidate has not been simply crammed for the occasion. To those who are opposed to the dental law I would say that it is better to have no law than to have good laws badly enforced. Everything in the law of the Province depends upon the men who form the Board of Examiners. If they are men without moral strength and without the welfare of the profession at heart, every kind of abuse and evasion of the law may be practised unchecked, and they may levy blackmail to their heart's content on the offenders. On the other hand, I think that the only way to elevate the standard of the profession as a whole is to enact rules with which all must comply before being allowed to practise. This is no real hardship to any one. It is simply a method of enforcing a certain standard of proficiency on those who wish to earn their living as dentists. It protects the public and the dental profession from quacks,—men whose chief stock-in-trade is impudence and ignorance.

The great defect of the dental law, as we have had it in Canada, has been that it has tended to make the dentists of each Province into a close corporation, whose chief effort has been to prevent men from outside from obtaining a right to practise in the Province, and in this way the real object of the law was lost sight of,—viz., the elevation and advancement of dentistry as a science. A brighter day has dawned at last, and we are beginning to open our eyes to the importance of a thoroughly good theoretical and practical education for our dental students.

Whether we advocate dental laws or not, we should all of us be anxious to assist to our utmost whatever tends to raise the standard of scientific knowledge and skill in our chosen profession.

